# CRISPR-mediated gene modification of hematopoietic stem cells with beta-thalassemia IVS-1-110 mutation

**DOI:** 10.1186/s13287-020-01876-4

**Published:** 2020-09-10

**Authors:** Hala Gabr, Mona Kamal El Ghamrawy, Abdulrahman H. Almaeen, Ahmed Samir Abdelhafiz, Aya Osama Saad Hassan, Maha Hamdi El Sissy

**Affiliations:** 1 Clinical and Chemical Pathology, Faculty of Medicine, Cairo University, Cairo, Egypt; 2 Pediatric, Faculty of Medicine, Cairo University, Cairo, Egypt; 3 Pathology Department, Jouf University, Sakakah, Saudi Arabia; 4 Clinical Pathology, National Cancer Institute, Cairo University, Cairo, Egypt

**Keywords:** Thalassemia, CRISPR/Cas-9, Reverse hybridization, Hemoglobin beta gene mutation, Hematopoietic stem cells

## Abstract

**Background:**

β-Thalassemias represent a group of genetic disorders caused by human hemoglobin beta (*HBB*) gene mutations. The radical curative approach is to correct the mutations causing the disease. CRISPR-CAS9 is a novel gene-editing technology that can be used auspiciously for the treatment of these disorders. The study aimed to investigate the utility of CRISPR-CAS9 for gene modification of hematopoietic stem cells in β-thalassemia with IVS-1-110 mutation.

**Methods and results:**

We successfully isolated CD34^+^ cells from peripheral blood of β-thalassemia patients with IVS-1-110 mutation. The cells were transfected with Cas9 endonuclease together with guide RNA to create double-strand breaks and knock out the mutation. The mutation-corrected CD34^+^ cells were subjected to erythroid differentiation by culturing in complete media containing erythropoietin.

**Conclusion:**

CRISPR/Cas-9 is an effective tool for gene therapy that will broaden the spectrum of therapy and potentially improve the outcomes of β-thalassemia.

## Background

Mutations involving the β-globin gene are the most common cause of genetic disorders in humans. β-Thalassemia constitutes a group of hereditary disorders affecting the expression of adult β-globin gene. They are caused by a few hundred of mutations decreasing or completely negating β-globin production. As a result, adult α2β2 hemoglobin (HbA) is reduced and excess α-globin content accumulates in the erythroid cells resulting in ineffective erythropoiesis and augmented apoptosis [20]. Besides being prevalent among Africans, β-thalassemias are also common in Greeks and Italians [30]*.* It has been estimated that 1000 children out of 1.5 million live births are born annually with thalassemia major in Egypt [11]. In Egyptian multi-centric studies, the carrier rate has been reported to be in the range of 9–10% [9]. The more than 350 β-thalassemia mutations reported are geographically widely diverse with a small number of specific mutations in individual populations. Thalassemia syndromes are common in Saudi Arabia. Beta-thalassemia gene mutations, particularly B^+^ and B^o^ thalassemia, occur with variable frequency in different regions of Saudi Arabia [6].

Thalassemia therapy constitutes a major socioeconomic burden. Various methods of treatment have been attempted, yet they have not been efficiently successful till now. Recently, the technology of clustered regularly interspaced short palindromic repeats (CRISPR)-based DNA editing has emerged as an encouraging tool to rectify genetic abnormalities in thalassemias [12]. Cas9 (CRISPR-associated protein-9 nuclease) is an RNA-guided endonuclease that utilizes RNA-DNA base pairing to spot target genomic DNA. Bound to its target via the guide RNA (gRNA), Cas9 generates DNA double-strand breaks (DSBs) at the pre-specified genomic sites that instantaneously activate the endogenous DNA repair mechanisms [22]. DSB repair is accomplished by either non-homologous end joining (NHEJ) or homology-directed repair (HDR; specific to S and G2 phases of dividing cells where sister chromatids exist). CRISPR/cas-9 genome editing system is a promising, feasible and safe approach for treating hemoglobinopathies or thalassemia given its efficiency [34].

The aim of this study was to assess the utility of CRISPR/Cas9 technology in correcting the *HBB* gene mutations in CD34^+^ cells collected from β-thalassemia patients with the Mediterranean cryptic splice site IVS-1-110 (G → A) mutation.

## Methods

### Patients

The study was conducted on six pediatric β-thalassemia patients with IVS-1-110 mutation. The patients’ custodians accepted to participate in the current study by signing a written consent. Patients were selected from Hematology Clinic of Abul-Reesh Mounira Children’s Hospitals, Cairo University, Cairo, Egypt. They were diagnosed as β-thalassemia by routine work-up and history including age, consanguinity, and blood transfusion. The research was approved by the ethical committee of the Clinical Pathology and the Pediatrics Departments, Kasr Alainy Hospitals, Cairo University.

Relevant demographic data of the patients were collected and a complete clinical examination was done with attention to the pallor, mongoloid faces, and the size of the liver and spleen. Investigations including complete blood count using an automated cell counter (Cell Dyne, USA), reticulocyte count using brilliant cresyl blue, Hb electrophoresis, and genotyping of β-thalassemia globin gene mutation by reverse hybridization technique were done for all patients.

### Methods

#### Separation of mononuclear cells from human peripheral blood

This separation was aseptically done by gradient centrifugation using Ficoll Hypaque according to the method of [39]. Briefly, heparinized blood samples were carefully suspended in 5 mL of 60% Ficoll Hypaque separation solution in a sterile conical tube, centrifuged for 20–25 min at 2000 rpm at 8 °C. The mononuclear cells were retrieved from the buffy coat layer by sterile Pasteur pipette, washed three times with phosphate-buffered saline (PBS), and centrifuged at 2000 rpm for 20 min. Cells were counted using automated cell counter (Cell Dyne, Inc., USA).

#### Magnetic separation of CD34^+^ cells from mononuclear cells with the auto-MACS separator

Cell population evaluation by the expression of CD34 marker was done as a functional assay to prove the cells are indeed HSC. Performing functional assays such as clonogenic ability (CFU formation in semisolid culture) can be done in a subsequent expansion of the work.

Cells were re-suspended in PBS, and anti-CD34 monoclonal antibody is added and mixed well followed by incubation for 10 min in the dark in the refrigerator. Then they were washed and resuspended in the buffer, and the anti-PE MicroBeads are added. Cells were subjected to magnetic separation on MACS separator.

#### Cas9 transfection, cell culturing, and induction of the erythroid differentiation

Cas9 protein bound to the guide RNA were non-virally transfected using the calcium phosphate method. Specified by the target DNA sequence, two *HBB* gene-based gRNA candidates were selected and the sequences of each gRNA candidate/name of the source plasmid are (1) GTAACGGCAGACTTCTCCTC**NGG/**HBB.g14 and CTTGCCCCACAGGGCAGTAA**NGG/** HBB.g13, respectively; the Proto-spacer Adjacent Motif (PAM) site is in bold.

#### Preparation of selective media and determining puromycin concentration

**Fig. 1:**
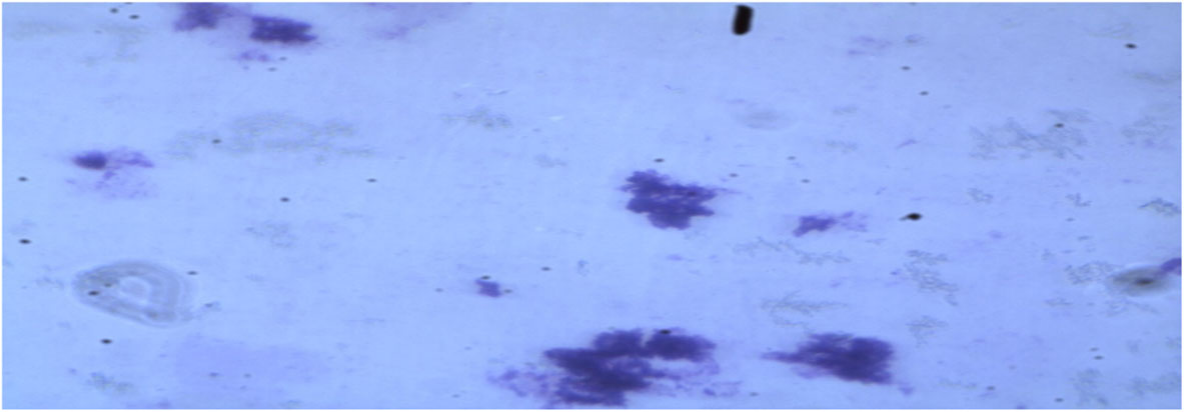
Functional assay of peripheral blood patient-derived hematopoietic stem cells (HSCs) under an inverted microscope at day 17 of culture differentiated into erythroid precursors (BFU-E colonies in semisolid cultures) under the effect of erythropoietin, trypan blue staining (high-power magnification × 40)

The kill dose-response curve was used to determine the antibiotic concentration sufficient to kill all of the non-transfected cells in 4 days duration. Calcium phosphate transfection [40] and erythroid culture were accomplished. Functional assay for erythroid progenitors was done:

- Morphology: appearance of BFU-E colonies in semisolid culture (Fig. 1)
- Hemoglobin synthesis: RNA extraction of beta-globin gene from cultured erythroid progenitors and further identification of the presence or absence of beta-globin mutations

Genomic DNA extraction using PureLink™ Genomic DNA Mini Kit was done according to the manufacturer’s instructions. This was followed by an evaluation of the β-globin gene product by PCR for normal and aberrant DNA (IVS-1-110) in the erythroid cultured cells.

#### Polymerase chain reaction (PCR)

All PCR reactions were done in 25 μL volume using: Master Mix (GeneON), forward (5^′^-TAATACGACTCACTATAGGG-3^′^) and reverse primers (5^′^-CCTCGACTGTGCCTTCTA-3^′^), and the extracted genomic DNA. The thermocycler program applied comprised initial denaturation at 94 °C for 2 min, 35 cycles of denaturation at 94 °C/15 s, annealing at 58 °C/30 s and extension at 72 °C/45 s, and a final extension at 72 °C/3 min.

#### Detection of β-globin gene mutation by PCR and reverse hybridization

PCR amplification products were detected using gel electrophoresis and were visualized on UV trans-illumination looking for bands with 310, 381, and 755 bp sizes. This was followed by detection by hybridization of amplification products to a test strip containing allele-specific oligonucleotide probes immobilized as an array of parallel lines (β-globin strip assay, Vienna Lab). The genotype of the sample was determined using the enclosed Collector™ sheet.

## Results

The relevant demographic data of the investigated β-thalassemia pediatric patients and their laboratory findings are presented in Table 1.

**Table 1:** Demographic and laboratory data of the investigated pediatric β-thalassemia patients

Variable	Case 1	Case 2	Case 3	Case 4	Case 5	Case 6
**Age (years)**	10 years	8 years	2 years	1.5 years	4 years	3 years
**Gender**	Male	female	Female	Male	Female	Male
**Duration of blood transfusion**	8 years	7 years	1.5 years	1 year	3 years	2.5 years
**RBCs (× 10**^**6**^**/mm**^**3**^**)**	3	3.2	3.8	4.1	3.2	4.6
**Hb (g/dL)**	8.5	9.2	9.5	10.1	9.4	9.2
**Hematocrit (%)**	28.9	30.2	30.9	30.4	29.5	30.2
**MCV (fL)**	70.5	69.6	71.4	65.4	72.4	74.6
**MCH (pg)**	25.4	22.4	25.6	24.6	26.1	25.9
**MCHC (g/dL)**	30.8	32.8	32.6	30.9	33.1	30.6
**RDW (%)**	18.6	17.5	16.9	17.5	18.4	18.6
**TLC (× 10**^**3**^**/mm**^**3**^**)**	15.5	20.6	14.6	17.6	14.9	25.6
**Platelets(× 10**^**3**^**/mm**^**3**^**)**	466	522	416	650	578	563
**Reticulocytes (%)**	6.5	5.4	10.2	8	4.5	5.5
**Serum iron (μg/dL)**	80	61	102	95	67	90
**HbF (%)**	80.2	84.6	74.02	66.4	70.2	88.4

CD34^+^ cells were isolated and evaluated. Table 2 presents the efficiency of the yield of CD34^+^ cells among the investigated patients. The highest yield of CD34^+^ cells was in case 4, in which there was near 94% CD34^+^ cell retrieval success.

**Table 2:** Percent retrieval of CD34+ cells from the investigated pediatric β-thalassemic patients

Case	CD34+ cells before separation		CD34+ cells after separation		Total mononuclear cell count	% retrieval of CD34+ cells
	Absolute count	Cells %	Absolute count	Cells %		
**Case 1**	1120	2.0	1000	90	56 × 10^3^	89.2%
**Case 2**	2700	3.2	2500	92	85 × 10^3^	92.5%
**Case 3**	1110	3.0	1000	87	37 × 10^3^	90%
**Case 4**	3210	2.2	3000	95	146 × 10^3^	93.4%
**Case 5**	2250	3.0	2000	90	75 × 10^3^	88.8%
**Case 6**	1300	2.0	1000	91	65 × 10^3^	77%

Determination of the antibiotic kill-dose response curve that showed a minimal concentration of antibiotic that efficiently killed all non-transfected/transduced cells after treatment for up to 6 days was found to be 12 μg/mL (Table 3).

**Table 3:** Dose-dependent percentage of non-transfected cell viability after treatment with different concentrations of puromycin for up to 6 days

Puromycin concentration	Non-transfected cell viability %
0 μg/mL	100
3 μg/mL	100
6 μg/mL	85
9 μg/mL	30
12 μg/mL	0
15 μg/mL	0

### Characterization of cultured erythroid cells

Differentiation of peripheral blood-derived CD34^+^ cells into erythroid precursors in serum-free medium was induced by erythropoietin (EPO). Starting from day 7, morphologically homogeneous erythroid progenitors were expanded into mass cultures in a quick time. Non-erythroid progenitors ceased their development secondary to the absence of necessary cytokines to support their proliferation and differentiation. On the other hand, erythroid progenitors were allowed to proliferate and differentiate into erythroid precursors under the effect of EPO (Fig. 1). Viability was assessed by calculating the percentage of viable cells after the addition of trypan blue vital stain.

Cell growth was quantitated by counting the cell number with a cell counter (Sysmex, Siemens). The maximum fold increase in proliferation, among the transfected cells, was observed for cells derived from case 2 (14-folds), whereas the least proliferation rate was recorded for cells derived from cases 1 and 6 (both 5-folds) (Table 4).

**Table 4:** Fold increase in cell number after erythroid liquid culture of transfected cells

Case	Initial input, cell/mL	Non-transfected cells after culture		Transfected cells after culture	
		Number	Fold increase	Number	Fold increase
**Case 1**	200	800	4	1000	5
**Case 2**	500	1000	2	7000	14
**Case 3**	200	800	4	2400	12
**Case 4**	600	1000	1.6	5000	8.3
**Case 5**	400	900	2.25	4500	11.2
**Case 6**	200	1000	5	1000	5

#### Molecular evaluation

##### PCR and gel electrophoresis

**Fig. 2:**
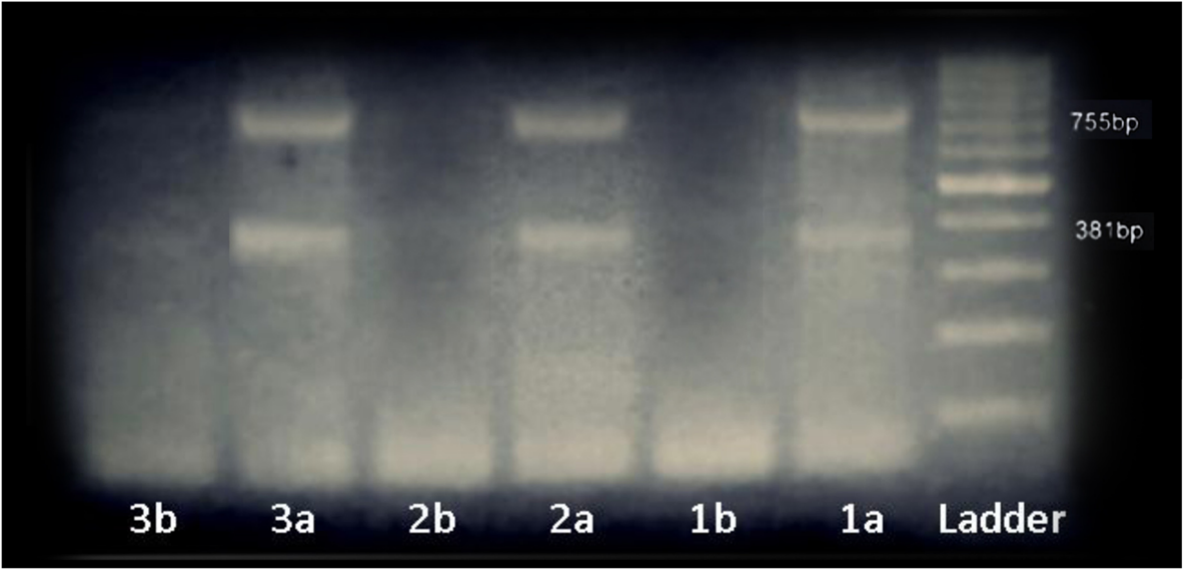
Gel electrophoresis of the HBB gene-amplified PCR products. Lanes 1a, 2a, and 3a represent CRISPR/Cas9 edited cells and show bands at 381 bp and 755 bp size which correspond to the target amplification product as instructed by the manufacturer protocol. Lanes 1b, 2b, and 3b represent the control unedited cells. They show no bands

PCR amplicons of the *HBB* gene of the CRISPR/Cas9-edited cells and the unedited cells were electrophoretically separated on 1.5% agarose gel. The target 381 bp and 755 bp size amplicons were observed only in the edited cells but not in the control unedited cells (Fig. 2).

##### Reverse hybridization

**Fig. 3:**
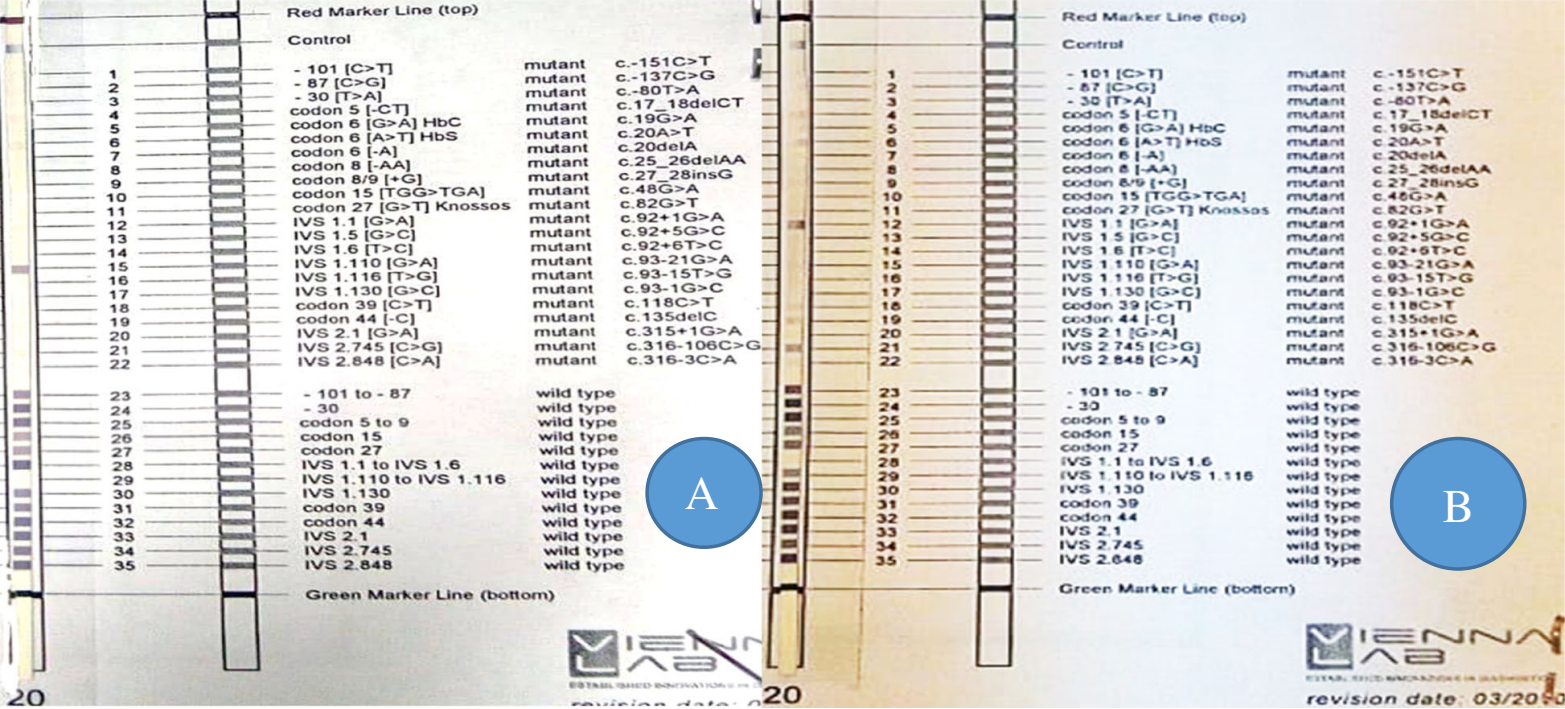
Reverse hybridization of a thalassemia patient before and after editing using CRISPR. **a** Reverse hybridization of unedited cells. IVS-1-110 mutation is shown. **b** Reverse hybridization of CRISPR/Cas9-edited cells. The IVS-1-110 mutation disappeared and the wild-type sequence was regained

PCR amplicons of the *HBB* gene of the CRISPR/Cas9 edited cells vs. the unedited control cells were subjected to reverse hybridization using β-globin strip to assess the presence of mutation. The mutation disappeared in the transfected cells that gained the wild-type gene sequence (Fig. 3).

##### Discussion

Thalassemias represent a heterogeneous group of inherited disorders that affect synthesis of the different globin chains [16]. According to a World Health Organization (WHO) report, “approximately 5% of the world’s population carries gene traits for hemoglobin disorders; mainly sickle cell anemia (SCA) and α- or β-thalassemias” [41]. The latter is one of the most prevalent hereditary diseases worldwide [2]. Genetic mutations causing β-thalassemia may lie within the β-globin gene or externally within the globin locus. Point mutations constitute the majority of genetic lesions implicated in the pathogenesis of β-thalassemia. In β-thalassemia, A few hundred mutations have been described affecting a wide range of gene processes that include transcription, mRNA splicing/posttranscriptional processing, RNA stability and translation, and globin peptide stability [16]. The splicing mutation in intron 1 of β-globin gene (IVS-1-110) is the most common mutation in Egyptian thalassemics, causing an aberrant splicing of pre-mRNA and deficient synthesis of β-globin chain [10].

Although the ultimate treatment for β-thalassemia is hematopoietic stem cell transplantation (HSCT), this option is only accessible to a minority of patients due to dearth of matched donors. Moreover, HSCT is a tough treatment associated with high morbidity [28] that has been ameliorated with the increasing use of non-myeloablative conditioning therapy [16].

Gene correction utilizing a patient’s autologous stem cells is considered a great progress that overcomes tissue matching and decreases the incidence of the associated morbidity and mortality from allogeneic HSCT [16]. CRISPR/Cas9 represents one of the most promising tools in gene editing [34]. Gene therapy of β-thalassemia can fall into one of 4 categories: (1) gene addition, (2) gene knockdown to improve the β-globinopathy phenotype, (3) globin gene editing, and (4) gene editing of globin regulatory elements [19]. Three different systems for specific DNA cleavage to promote efficient homologous recombination have been introduced: ZFNs, TALENs, and CRISPR/Cas9 [42]. ZFNs are demanding in terms of design and cost. TALENs necessitate making numerous pairs to test their cutting efficiencies. In addition, DNA methylation and histone acetylation may affect their efficiency [23, 26, 31, 33]. In comparison, the CRISPR/Cas9 is not amenable to those limitations, feasible, and easily prepared [21].

This study assessed the utility of CRISPR/Cas9 in directly targeting IVS-1-110 mutation site in *HBB* locus, which prevails among β-thalassemic Egyptians. Our findings support the prospect of β-thalassemia gene correction using such a promising technology. The study included 6 patients diagnosed with β-thalassemia selected from the Hematology Clinic, Abou-El-Reesh-Mounera Children’s Hospitals, Cairo University, with homozygous IVS-1.110 mutation as diagnosed by reverse hybridization using β-globin strip assay (Vienna Lab). Peripheral blood CD34^+^ hematopoietic stem cells were isolated by MACS method with high purity and yield of more than 90%. The presence of microbeads at the surface of the separated CD34^+^ cells did not appear to have any negative influence on hematopoietic recovery, proliferation, homing, or the side-effects following re-infusion into patients. These findings are in agreement with [8] and Traxler et al. [36], who obtained and enriched CD34^+^ cells by immunomagnetic bead selection using an Auto-MACS technique (Miltenyi Biotec, Germany). Tomlinson et al. [35] reported that MACS separation is quicker than FACS straining, where a greater number of cells can be processed at one time. MACS does not require a rather expensive dedicated instrumentation. On the downside, multi-marker selection and simultaneous sorting of different populations are limitations of the MACS method [27, 29].

Compatible with the protocol done by Traxler et al. [36], our study adopted the three-phase erythroid differentiation protocol of CD34^+^ hematopoietic stem and progenitor cells (HSPCs): phase 1 (days 0–7), phase 2 (days 8–12), and phase 3 (≥ 13 days). We successfully transfected the isolated CD34^+^ cells with gRNA/Cas9 complex non-virally using the calcium phosphate transfection method. This avoids the viral immunogenicity and cytotoxicity, with relative feasibility and higher DNA carrying capacity [1]. Actually, numerous previous studies used the inorganic calcium as a non-viral vector for nucleic acid transfection [15, 24, 25].

In our study, the CRISPR/Cas9-edited cells showed the acquisition of the wild-type gene at IVS-1.110 position which was absent in the untreated control cells, although another region of the gene was affected at IVS-1-1 locus due to the natural high rate of indel formation associated with NHEJ repair. Coetzer [3] showed that β-globin gene correction through HDR in CD34^+^ HSPCs using CRISPR/Cas9 and a wild-type gene donor sequence is relatively inefficient since these cells are quiescent—as HDR process is restricted to S and G2 phases of the cell cycle when sister chromatids are available as repair templates. In addition, our results are in agreement with Genovese [14] who concluded that HSCs are more resistant to HDR-mediated editing. However, homology-mediated end joining may be facilitated by the predominance of 13-nt deletion resulted from the cleavage site of Cas9 when flanked by 8-nt tandem repeats [37]. The use of gRNA for the normal template may overcome this obstacle by inducing HDR which inserts the desired sequences through recombination of the target locus with exogenously supplied DNA donor templates [32].

Similar to previous pioneering reports, our study confirmed the utility of CRISPR/Cas9 gene editing to correct mutations that cause hemoglobinopathies and thalassemias. They showed that CRISPR/Cas9 system could correct *HBB* gene mutations in cells isolated from hemoglobinopathic/thalassemic patients ([4, 5, 7, 13, 18, 21, 17]). The edited HSPCs that differentiate into erythrocytes express wild-type mRNA from the endogenous *HBB* promoter [38].

## Conclusion

CRISPR/Cas9 system is a promising tool for the next-generation gene-editing therapy of thalassemias. In this work, the CRISPR-Cas9 system was able to specifically target the HBB gene sequence and cleavage it. This promoted efficient homologous recombination and acquisition of wild-type HBB mRNA in the transfected cells. Our results represent an addition to the existing evidence for the utility of this as a radical treatment for thalassemias and hemoglobinopathies. More studies should be carried out to refine the gene-editing process and to minimize potentially harmful off-target mutations before human clinical trials are considered.

## Data Availability

All data generated or analyzed during this study are included in this published article.

## Abbreviation

CRISPR: Clustered regularly interspaced short palindromic repeats
DNA: Deoxyribonucleic acid
DSB: Double-strand breaks
EPO: Erythropoietin
HbA: Adult hemoglobin
Hb: Hemoglobin
HBB: Hemoglobin B
HDR: Homology-directed repair
HSCPc: Hematopoietic stem and progenitor cells
HSCT: Hematopoietic stem cell transplantation
IVS: Intervening sequence
MACS: Magnetic activated cell sorting
MCH: Mean corpuscular hemoglobin
MCHC: Mean corpuscular hemoglobin concentration
MCV: Mean corpuscular volume
NHEJ: Non-homologous end joining
ORF: Open reading frame
PAM: Proto-spacer Adjacent Motif
PBS: Phosphate-buffered saline
PCR: Polymerase chain reaction
RBCs: Red blood cells
RDW: Red cell distribution width
RNA: Ribonucleic acid
SCA: Sickle cell anemia
TALEN: Transcription activator-like effector nuclease
TLC: Total leucocytic cells
WHO: World Health Arganization
ZFN: Zinc finger nuclease
